# Chemokine ligand–receptor interactions critically regulate cutaneous wound healing

**DOI:** 10.1186/s40001-017-0299-0

**Published:** 2018-01-16

**Authors:** Erich Bünemann, Norman-Philipp Hoff, Bettina Alexandra Buhren, Ulrike Wiesner, Stephan Meller, Edwin Bölke, Anja Müller-Homey, Robert Kubitza, Thomas Ruzicka, Albert Zlotnik, Bernhard Homey, Peter Arne Gerber

**Affiliations:** 1 Department of Dermatology, University Clinic Duesseldorf, 40225 Duesseldorf, Germany; 2 Department of Radiation Oncology, University Clinic Duesseldorf, 40225 Duesseldorf, Germany; 3 Department of Dermatology, Ludwig-Maximilian-University of Munich, 80539 Munich, Germany; 4 Department of Biology, Senomyx, Inc, 4767 Nexus Center Drive, San Diego, CA 92121 USA

**Keywords:** Chemokines, Chemokine receptors, Wound healing, Skin, Keratinocyte, Fibroblast, Endothelial cell, Leukocyte

## Abstract

**Background:**

Wound healing represents a dynamic process involving directional migration of different cell types. Chemokines, a family of chemoattractive proteins, have been suggested to be key players in cell-to-cell communication and essential for directed migration of structural cells. Today, the role of the chemokine network in cutaneous wound healing is not fully understood. Unraveling the chemokine-driven communication pathways in this complex process could possibly lead to new therapeutic strategies in wound healing disorders.

**Methods:**

We performed a systematic, comprehensive time-course analysis of the expression and function of a broad variety of cytokines, growth factors, adhesion molecules, matrixmetalloproteinases and chemokines in a murine cutaneous wound healing model.

**Results:**

Strikingly, chemokines were found to be among the most highly regulated genes and their expression was found to coincide with the expression of their matching receptors. Accordingly, we could show that resting and activated human primary keratinocytes (CCR3, CCR4, CCR6, CXCR1, CXCR3), dermal fibroblasts (CCR3, CCR4, CCR10) and dermal microvascular endothelial cells (CCR3, CCR4, CCR6, CCR8, CCR9, CCR10, CXCR1, CXCR2, CXCR3) express a distinct and functionally active repertoire of chemokine receptors. Furthermore, chemokine ligand–receptor interactions markedly improved the wound repair of structural skin cells in vitro.

**Conclusion:**

Taken together, we here present the most comprehensive analysis of mediators critically involved in acute cutaneous wound healing. Our findings suggest therapeutic approaches for the management of wound closure by targeting the chemokine network.

**Electronic supplementary material:**

The online version of this article (10.1186/s40001-017-0299-0) contains supplementary material, which is available to authorized users.

## Background

Wound healing is a complex phenomenon that requires an integrated network of repair mechanisms, including cell migration, proliferation and cellular production of structural molecules essential for tissue regeneration. The tissue response to injury is characterized by three overlapping phases: (a) Inflammation, (b) granulation and (c) matrix remodeling [1]. These phases involve a dynamic cascade of events including clotting and hemostasis, leukocyte recruitment, tissue formation, epithelialization, angiogenesis, collagen synthesis and wound contraction [1]. The major cell types involved in cutaneous wound healing span from hematopoietic cells such as blood platelets, immune regulators like neutrophils, T cells, dendritic cells and macrophages, to structural cells like fibroblasts, endothelial cells and keratinocytes [1–6]. Specifically, wound repair is regulated by a highly orchestrated interplay of cytokines, growth factors, extracellular matrix and more recently described chemokines [2, 3, 6–15]. Chemokines and their receptors play a crucial role in development, homeostasis, tumor development and most notably wound healing and angiogenesis [16–18].

Chemokines are small, secreted proteins which mediate directional migration and regulate leukocyte trafficking [19–21]. Notably, the chemokine family is likely to be one of the first complete protein superfamilies that has been identified and characterized at molecular level [22]. This presents the opportunity to identify all relevant members of the chemokine superfamily involved in complex biological processes such as wound healing.

The aim of this work was to investigate the complex interactions of chemokines and their receptors in cutaneous wound healing. We systematically measured cytokines, growth factors, adhesion molecules, matrixmetalloproteinases and chemokines in a murine model for acute cutaneous wound healing. Strikingly, a subset of chemokines proved to be among the most highly regulated genes. The chemokine expression is highly orchestrated and coincides with the appearance of matching receptors. Accordingly, we demonstrate that structural cells, such as human keratinocytes, dermal fibroblasts and dermal microvascular endothelial cells, express a distinct and functionally active repertoire of chemokine receptors. Furthermore, migration assays suggest that chemokines and their cognate receptors accelerate wound healing via chemotaxis of structural cells.

## Methods

### Cell culture

Human primary epidermal keratinocytes, dermal fibroblasts, melanocytes and dermal microvascular endothelial cells were purchased from Clonetics (San Diego, CA) and cultured in keratinocyte (KGM-2), fibroblast (FGM-2), or endothelial cell (EGM-2) growth medium (all Clonetics, San Diego, CA) [23]. We have used 2 × 10^4^ cells for the culture and let it grow up to 80% confluence. Cells were treated with TNF-α (10 ng/ml)/IL-1β (5 ng/ml) (R&D Systems Inc., Minneapolis, MN) for 10 to 12 h or left untreated. RNA was extracted from cells immediately, after 8 and 18 h as described later.

### Quantitative real time PCR analysis

Quantitative real-time PCR analyses were performed as previously described [21, 24, 25]. Skin biopsies were homogenized in liquid nitrogen and RNA was extracted with RNAzol according to the manufacturer’s protocol (Tel-Test, Friedensburg, TX). 4 μg of RNA was reverse transcribed using standard protocols. 25 ng of cDNA was amplified in the presence of 12.5 μl of TaqMan^®^ universal master mix (Applied Biosystems, Foster City, CA), 0.625 μl of gene-specific TaqMan^®^ probe, 0.5 μl of gene-specific forward and reverse primers, and 0.5 μl of water. As an internal positive control, 0.125 μl of 18S RNA-specific TaqMan^®^ probe and 0.125 μl of 18S RNA-specific forward and reverse primers were added to each reaction. Gene-specific probes used FAM™ as reporter dye, whereas probes for the internal positive control (18S RNA) were associated with the VIC™ reporter dye. Alternatively, 25 ng of cDNA was amplified using target-specific primer combinations and SYBR green master mix (Applied Biosystems, Foster City, CA). Chemokine ligand- and receptor-specific primers and target-specific probes were obtained from Applied Biosystems (Foster City, CA). Gene-specific PCR products were measured by means of an ABI PRISM^®^ 7700 or 5700 Sequence Detection Systems (Applied Biosystems, Foster City, CA) continuously during 40 cycles. Target gene expression was normalized between different samples based on the values of the expression of the internal positive control (18S RNA) or ubiquitin.

### Flow cytometry

To analyze chemokine receptor expression of non-hematopoietic cells, cultured primary epidermal keratinocytes, dermal fibroblasts and dermal microvascular endothelial cells from different donors were analyzed using flow cytometry and the following monoclonal antibodies (mAb): PE-conjugated anti-CCR1 (53,504.111, IgG2β), CCR2 (48,607.211, IgG2β), CCR3 (61,828.111, IgG2α), CCR7 (150,503, IgG2α), CCR9 (112,509, IgG2α), CXCR4 (12G5, IgG2α), CXCR5 (51,505, IgG2α), CXCR6/STRL33 (56,811, IgG2β) all R&D Systems, Mineapolis, MN. CCR4 (1G1.1, IgG1), CCR5 (2D7/CCR5, IgG2α), CCR6 (11A9, IgG1) CXCR1 (5A12, IgG2β), CXCR2 (6C6, IgG1), CXCR3 (1C6/CXCR3, IgG1) all Pharmingen, San Diego, CA, CCR8 (Goat, IgG) Alexis Biochemicals, Grünberg, Germany, CCR10 (1908, IgG1) DNAX Research Inc., Palo Alto CA (or appropriate isotype controls (Pharmingen, San Diego, CA and R&D Systems, Mineapolis, MN, Jackson ImmunoResearch Laboratories, Inc., West Grove, PA, USA). Briefly, 10^6^ cells were stained with anti-chemokine receptor mAb or isotype and analyzed using a FACScan and CELLQuest software (Becton Dickinson, San Jose, CA).

### In vitro wound repair assay

2 × 10^4^ human primary dermal fibroblasts, 2 × 10^4^ dermal microvascular endothelial cells and 2 × 10^4^ epidermal keratinocytes were cultured in 24-well plates in FGM-2, EBM-2 and KGM-2 (Clonetics, San Diego, CA) medium and grown to confluency analogously to the previously described cultivation. The medium was removed and a single path wound was made with a sterile pipette tip through the intact cell layer. The width of the wound was 200 µm. Medium supplemented with or without CCL1/I-309, CCL11/eotaxin, CCL17/TARC, CCL20/MIP-3α, CXCL8/IL-8, CXCL9/MIG, CXCL10/IP-10 for keratinocytes, CCL1/I-309, CCL11/eotaxin, CCL17/TARC, CCL27/CTACK for dermal fibroblasts and CCL1/I-309, CCL11/eotaxin, CCL17/TARC, CCL20/MIP-3α, CCL27/CTACK, CXCL8/IL-8, CXCL9/MIG, CXCL10/IP-10 for dermal microvascular endothelial cells was added to each well. To confirm the involvement of G(i) alpha protein-coupled receptor signaling in chemokine-induced wound repair responses, structural cells were pre-incubated with or without pertussis toxin (100 ng/ml PTX, Calibochem, Germany) 10–12 h before injury and chemokine stimulation. The status of the single path wound was determined immediately as well as 8 and 12 h after the wounding using a digital camera system (Olympus, Hamburg, Germany), or was monitored for 12–18 h by time lapse video microscopy system (inverse Leitz Microscope with cell culture equipment, Zeiss AxioCam HRc, Zeiss AxioVision Software, Carl Zeiss, Oberkochen, Germany).

### Cutaneous wound healing model

A total of 27 (3 mice per defined time point) female BALB/c mice were anesthetized with an intraperitoneal injection of ketamine (120 mg/kg bw) and xylazine (16 mg/kg bw). The lumbar region was shaved and the surgical area was disinfected with betadine and isopropanol. Full-thickness 2 cm dorsal incisions were made with a scalpel through the epidermis and dermis leaving the subcutaneous muscle layer undisturbed. Wounds were closed using a single 5 W surgical staple. Mice were euthanized by CO_2_ inhalation 6, 12, 18 h, 1, 2, 3, 5, 7, and 10 days after incisions were done. At the time of harvest, dorsal pelts surrounding the incisions were removed and normal skin was trimmed to 1–2 mm of incision. One half of each sample was placed in 10% buffered formalin and processed for paraffin embedding. The other half of each sample was snap frozen in liquid nitrogen and processed for RNA extraction. Each cohort consisted of three animals and the subsequent experiment was repeated twice.

All experiments with mice were carried out after examination by the local ethics committee of the University of Düsseldorf and after an internal identification number was given. The tests were carried out by trained personnel, who respect the internal and international guidelines for animal research (e.g. ARRIVE Guidelines).

### Patients

This study was approved by the local ethics committee and informed consent was secured from all patients participating in this study. A total of 4 patients were included in the study. Skin biopsies were taken from either healthy individuals or patients undergoing surgery and secondary wound healing. Prior, 24 h, 2, 3, 5, 7, 9 and 12 days after radical surgery for acne inversa, biopsies were taken from the non-lesional wound edges. Skin samples were immediately frozen in liquid nitrogen and stored at – 80 °C.

### Histology and immunohistochemistry

Frozen skin samples, cut to a thickness of 6 µm on a cryomicrotome, were used for immunohistochemical analyses of human secondary wound healing, skin sections were fixed with 3% acetone and preprocessed with H_2_O_2_ followed by an avidin and biotin blocking step (VECTOR Blocking Kit). Sections were stained with monoclonal antibodies against CCR2α, CCR2β, CCR3, (all polyclonal goat IgG), CCR4 (rabbit IgG), CCL1/I-309, CCL11/eotaxin, CCL24/eotaxin-2 (goat IgG) (all Santa Cruz Biotechnology Inc., Santa Cruz, CA, USA), CD31 (JC/70A, mIgG1), CD68 (KP1, mIgG1) (DAKOCytomation, Hamburg, Germany), CCL4/MIP-1β, CCL17/TARC, CCL18/DC-CK1, CCL20/MIP-3α, CCL27/ALP (124,302.11; mIgG2α), CXCL9/MIG, CXCL10/IP-10 (all goat IgG), CXCL12/SDF-1α (K15C; mIgG1; Unite d’Immunologie Virale, Institute Pasteur, Paris, France), CCR1 (53,504.111; IgG2β), CCR5 (45,549.111; mIgG2β), CCR6 (53,103.111; mIgGβ), CXCR1 (42,705.111; mIgG2α), CXCR2 (48,311.111; mIgG2α), CXCR3 (49,801.111; mIgG1), CXCR4 (44,716.111; mIgG2α), CXCL10/IP-10, CXCL9/MIG, CCL17/TARC (all goat IgG) all R&D Systems, Minneapolis, MN), CXCL8/IL-8 (B-K8; mIgG2α) (Diaclone Res., France), CCR8 and CCR10 (German Cancer Research Center, Heidelberg, Germany) at 4oC. The staining was performed with an ABC-Kit (VECTOR Vectastain ABC-Kit) and an ACE-Kit (VECTOR). Sections were counterstained with hematoxylin. The immunohistochemical examinations were conducted by three independent pathologists. If there were differences in the evaluation, a joint result was finally established.

## Results

### The dynamic network of chemokines in murine cutaneous wound healing

**Fig. 1:**
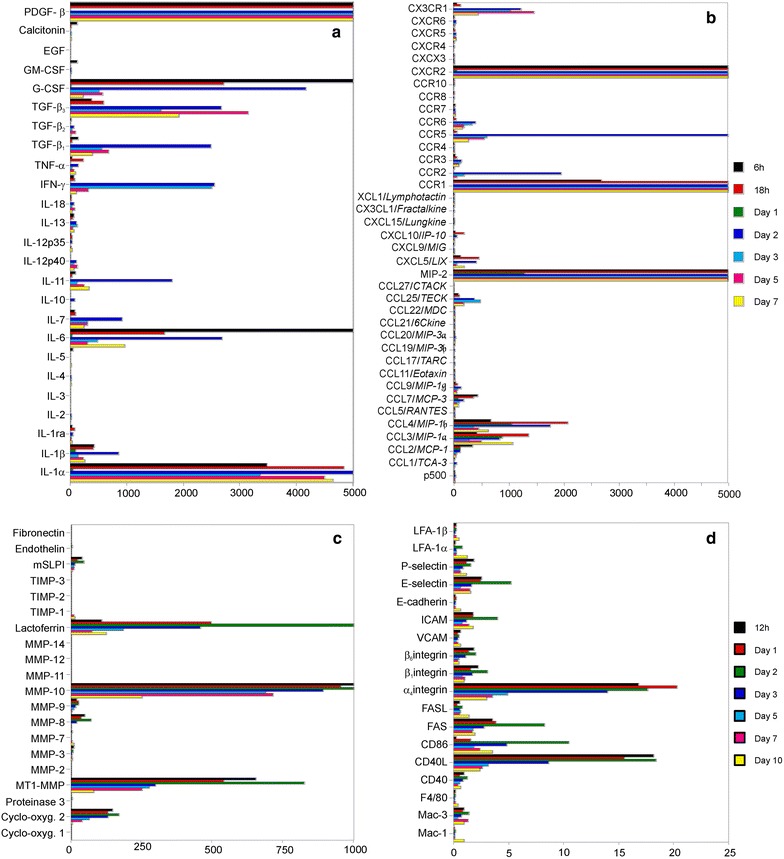
Differential expression of more than 100 genes involved in wound healing. A subset of chemokines is among the most highly regulated genes in wound healing. Quantitative real-time RT-PCR analyses of **a** cytokine, **b** chemokine, **c** matrix-metalloproteinase (MMP) and **d** adhesion molecule expression in cutaneous wound healing. Skin specimens of BALB/c mice were taken before or 6, 12, 18 h, 1, 2, 3, 5, 7, or 10 days after full thickness skin incision. Target gene expression is shown as fold increase compared to healthy skin. Representative data of two independent experiments

To elucidate the dynamic network of mediators controlling the highly orchestrated process of cutaneous wound healing, we performed comprehensive real-time PCR analyses of more than 100 genes including cytokines, growth factors, adhesion molecules, matrix-metalloproteinases and chemokines in a murine model of cutaneous wound healing (Fig. 1).

Based on the cytokines studied, along with IL-6 the IL-1 family members IL-1α and IL-1β showed the highest-fold upregulation after skin injury. IL-6 and TNF-α exhibited early peaks of expression after 6 or 18 h while IL-1 family members and IFN-γ, IL-7, IL-11 showed maximal upregulation at days 2–3 (Fig. 1a). Among growth factors, platelet-derived growth factor (PDGF)-β, transforming growth factor (TGF)-β and granulocyte-colony stimulating factor (G-CSF) showed highest fold-increases in mRNA expression (Fig. 1a). The maximum expression of members of the growth factor superfamily was observed during the transition from the inflammatory to the tissue formation phases at days 2–5. Within the family of matrix-metalloproteinases, MT1-MMP, MMP-10 (stromelysin-2), lactoferrin, MMP-9 and MMP-8 showed the highest fold upregulation (Fig. 1c). Several members of the integrin/adhesion molecule families were upregulated following skin injury with α4 and β7 integrins showing the most prominent upregulation (Fig. 1d). However, this may be misleading since some members of the integrin superfamily such as β1 integrin exhibit significant homeostatic expression in skin that translates into a lower fold-upregulation following injury.

Among all genes studied, the members of the chemokine superfamily are belonging to the highly regulated transcripts during cutaneous wound healing. In particular, the CC chemokines CCL3/MIP-1α and CCL4/MIP-1β were upregulated more than 1000-fold in injured compared to normal skin (Fig. 1b). Next to the chemokine ligands also significant increases of transcripts of their matching receptors (CXCR2, CCR1, CCR5) could be detected (Fig. 1b). The temporal kinetics of mRNA expression underscored the tightly regulated and orchestrated process of wound healing.

Three distinct expression patterns were recognized among the chemokines. First, chemokines which reached maximal upregulation within hours after initial trauma and subsequently disappeared during the following days (MIP-2, CCL3, CCL4, CCL2/MCP-1, CCL7/MCP-3, CCL11/eotaxin, CXCL10/IP-10, XCL1/Lymphotactin, CCR1, CXCR2) (Fig. 1b); second, members of this family of chemoattractive proteins which showed early upregulation but peak expression between days 3 and 5 (CCL5/RANTES, CXCL9/Mig, CXCL12/SDF-1, CCR2, CCR3, CCR5, CXCR3, CXCR4); third, chemokines which were overexpressed during the tissue formation and remodeling phase of cutaneous wound healing (> day 5; CCR6, CX3CR1). Notably, chemokine receptors closely followed the expression of their matching ligands (e.g. MIP-2-CXCR2, CCL3-CCR1, CCL5-CCR5).

**Fig. 2:**
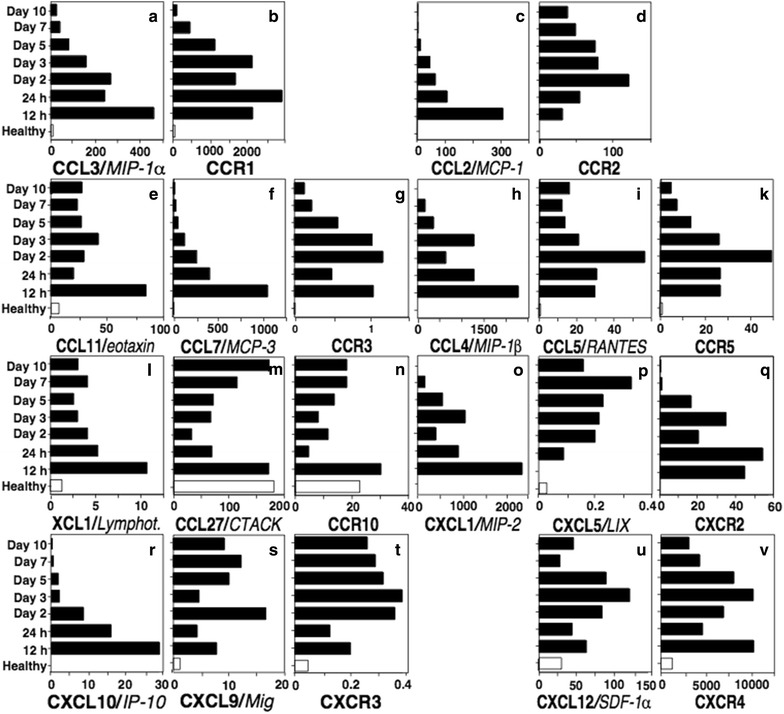
The chemokine and chemokine receptor expression is temporally regulated during cutaneous wound healing. Quantitative real-time PCR analyses of absolute levels of chemokine gene expression in cutaneous wound healing (**a**–**v**). Skin specimens of BALB/c mice were taken before or 12, 24 h, 2, 3, 5, 7, or 10 days after full thickness skin incision. Absolute values of gene expression were expressed as fg of target gene in 25 ng of total cDNA

Since fold-difference of expression does not indicate the abundance of transcripts present at a particular site, we additionally studied the absolute expression levels of target chemokines and their receptors. Absolute mRNA levels determined using real-time quantitative RT-PCR showed that MIP-2, CCL3 and CCL4 were not only the most highly regulated chemokines during cutaneous wound healing, but also showed the highest abundance of transcripts during the inflammatory phase of the healing process (Fig. 2a, h, o). Notably, their receptor expression mirrored the expression kinetics of the ligands (Fig. 2b, k, q). Next to the highly regulated chemokines MIP-2, CCL3 and CCL4, other chemokine ligands and their receptors also showed interesting expression patterns. CXCR3 was upregulated within hours after incision, showing peak expression between days 2 and 3 and remained upregulated until day 10 (Fig. 2t). While the CXCR3 ligand CXCL10 showed early and rapid upregulation within hours after injury, another CXCR3 ligand, CXCL9, showed delayed induction and persisting upregulation during cutaneous wound healing. Similar patterns could be observed for CCR2, CCR3 and CCR5 ligands (Fig. 2c, e, f, h, i). CCL2 showed a peak upregulation 12 h after incision, whereas the maximum of the corresponding CCR2 expression was reached at day 2. While CXCL12 and CXCR4 already showed a significant homeostatic expression in the skin, this ligand–receptor pair was still upregulated in an equivalent fashion reaching a peak at day 3 (Fig. 2u, v).

### Chemokine receptor profile of structural cells: keratinocytes, fibroblasts and endothelial cells

As mentioned above, structural cells such as keratinocytes, fibroblasts and endothelial cells undergo substantial migration processes during wound healing. Observations made in the mouse imply that chemokine ligand–receptor interactions play an important role in cutaneous wound healing. Therefore, we next sought to systematically characterize the chemokine receptor expression panels (CCR1-CCR10, CXCR1-CXCR6) of structural cells of the human skin in further detail (Table 1; Additional file 1: Figure S1–3). Since primary cytokines such as IL-1-family members and TNF-α are induced during the wound healing process, a cocktail of IL-1β plus TNF-α was used to mimic the stimulatory microenvironment during early cutaneous wound healing. Using flow cytometric analyses, we found that cultured human primary resting and IL-1β/TNF-α-activated keratinocytes showed distinct expression of CCR4, CCR6, CCR9, CXCR1 and CXCR3 as well as low levels of CCR3 (Table 1; Additional file 1: Figure S1), while dermal fibroblasts expressed CCR3, CCR4 and CCR10 on their cell surface (Table 1; Additional file 1: Figure S2). Cultured human primary dermal microvascular endothelial cells also showed a distinct pattern of chemokine receptors on their cell surface including CCR3, CCR4, CCR6, CCR8, CCR9, CCR10, CXCR1, CXCR2, and CXCR3 (Table 1; Additional file 1: Figure S3). No significant change in regulation was detected in cultured structural cells 12–24 h after IL-1β/TNF-α activation. As a trend, cytokine activation appeared to downmodulate rather than increasing cell surface expression of chemokine receptors. It is important to note that there was some degree of variability in the levels of chemokine receptor expression depending on different donors and the relative confluence of primary cell cultures; however, the overall picture of cell surface expression was not affected.

**Table 1:** Chemokine receptors expressed by cultured human primary keratinocytes, dermal fibroblasts and dermal microvascular endothelial cells

	CCR										CXCR					
	1	2	3	4	5	6	7	8	9	10	1	2	3	4	5	6
Keratinocytes	–	–	+	+	–	+	–	–	+	–	+	–	+	–	–	–
Fibroblasts	–	–	+	+	–	–	–	–	–	+	–	–	–	–	–	–
Endothelial cells	–	–	+	+	–	+	–	+	+	+	+	+	+	–	–	–

Flow cytometry was performed with PE-labeled anti-chemokine receptor mAbs directed against CCR1 to CCR10 and CXCR1 to CXCR6 or specific isotype controls using cultured primary cells. Representative results of three different donors

### Regulation of relevant chemokine ligands in structural cells

**Fig. 3:**
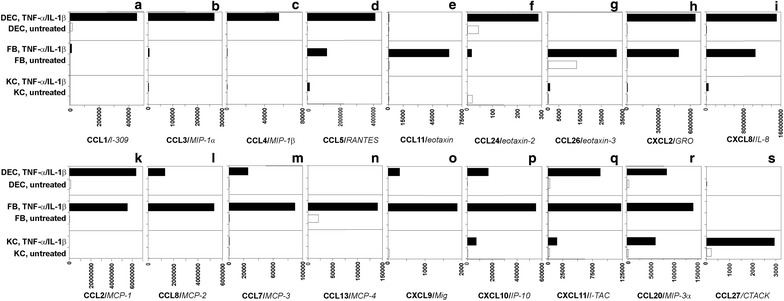
Chemokine ligands are differentially regulated in resting and TNF-α/IL-1β-activated keratinocytes, dermal fibroblasts and dermal microvascular endothelial cells (**a-s**). Quantitative real-time PCR analyses of absolute levels of chemokine gene expression of resting and activated (TNF-α, IL1-β) human primary keratinocytes (KC), dermal fibroblasts (FB) and dermal microvascular endothelial cells (DEC). Absolute values of gene expression were expressed as fg of target gene in 25 ng of total cDNA

Chemokine receptors and their respective ligands are differentially regulated in a highly coordinated fashion during murine cutaneous wound healing. Accordingly, we have demonstrated that resting as well as activated structural cells of the human skin express distinct patterns of chemokine receptors. To now identify the putative cellular origin and the regulatory patterns of the respective chemokine ligands in human skin cells, we next preformed systematic quantitative real-time PCR analyses in resting and TNF-α/IL-1β-activated keratinocytes, dermal fibroblasts and dermal microvascular endothelial cells. Strikingly, an array of chemokines showed homeostatic expression in structural cells, i.e. CCL20/MIP-3α and CCL27/CTACK in keratinocytes, CCL13/MCP-4 and CCL26/eotaxin-3 in dermal fibroblasts and CCL1/I-309 and CCL24/eotaxin-2 in dermal microvascular endothelial cells (Fig. 3). In vitro, the MCP-family members CCL2, CCL7, CCL8/MCP-2, and CCL13 as well as CCL11, CCL26 were markedly induced by IL-1β/TNF-α in cultured dermal fibroblasts (Fig. 3). Interestingly, analyses of structural cells of the skin suggested that CCL13 expression is restricted to dermal fibroblasts (Fig. 3). CCL1, CCL5, CCL3, CCL4, CXCL2/GRO and CXCL8 showed dominant induction in dermal microvascular endothelial cells. Notably, CXCL8 showed high expression in dermal microvascular endothelial cells but not in cultured keratinocytes (Fig. 3).

In vitro, TNF-α/IL-1β-stimulation resulted in marked upregulation of the CXCR3 ligands CXCL9, CXCL10, and CXCL11 in endothelial cells, fibroblasts and to a lower degree in keratinocytes.

Keratinocytes constitutively produced high levels of CCL20 and CCL27 transcripts which were highly-inducible by primary proinflammatory cytokines.

### Chemokines mediate wound repair of structural cells in vitro

**Fig. 4:**
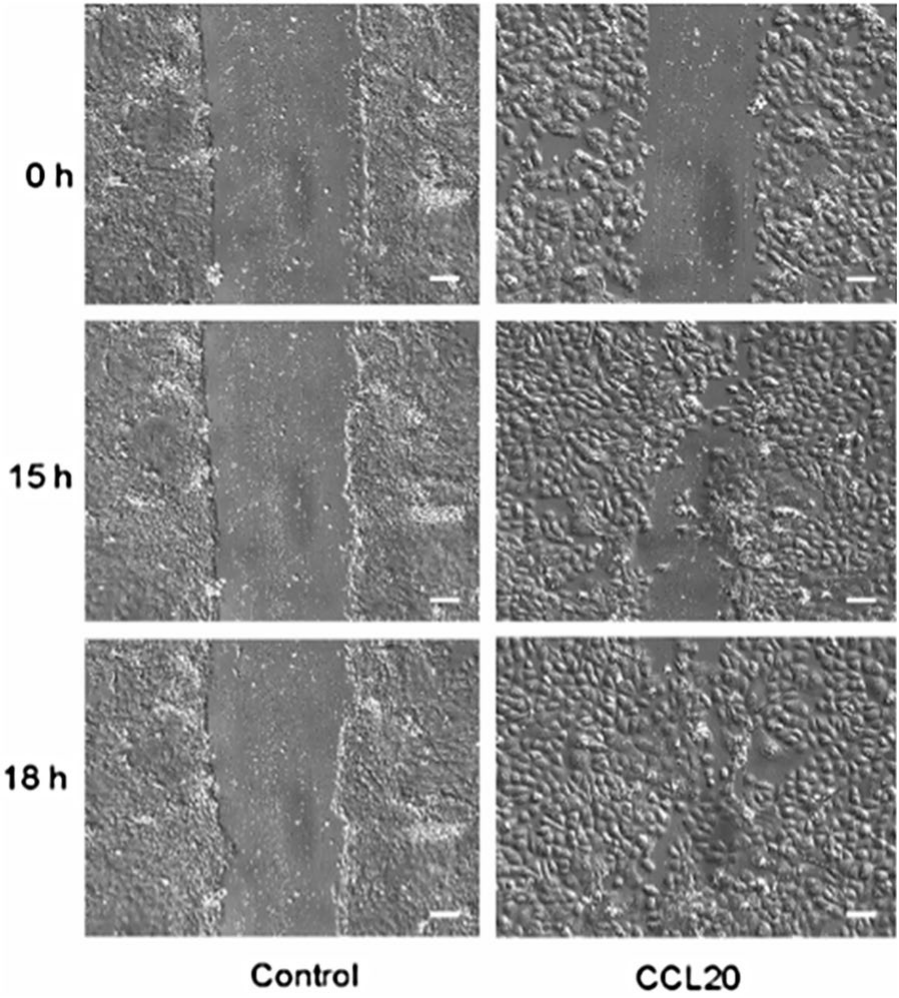
Chemokines enhance wound repair in vitro. Wound repair of a single path wound in a confluent culture of human primary keratinocytes, in the presence of chemokine CCL20 or medium alone (control) was monitored by time-lapse video microscopy for 18 h after injury. 0 h, monolayers immediately after injury; 18 h, keratinocyte monolayer with CCL20/MIP-3α (100 ng/ml) was nearly closed compared to control. Magnification ×150. The scale corresponds to 50 µm. Representative results of at least three independent experiments

To now test the functional implications of our expression analyses, we next sought to perform in vitro wound repair assays. Therefore, we studied the effects of relevant chemokines on primary keratinocytes, dermal fibroblasts and dermal microvascular endothelial cells in assays which were designed either as “end-time”-Assay or as continuously monitored assay with timelapse video microscopy (Fig. 4). Briefly, structural cells were grown to confluency and single path wounds were generated. As wound injury repair is mediated by migration within the first 24 h and cell proliferation occurs later than 36 h, wound closure was monitored and determined 12–18 h after injury.

Cultured keratinocytes express CXCR1, CXCR3, CCR4, CCR6 and CCR9 on their cell surface. The strongest chemokine-induced wound repair of keratinocytes could be achieved with the CCR6 ligand CCL20 and the CXCR3 ligand CXCL10 with dose-dependent closure of single path wounds within 12 h (Table 2, Fig. 4). Weaker but significant responses could be observed for CXCL8, a CXCR1 ligand, and CCL11, which binds to CCR3. However, stimulation of keratinocytes with the CCR4 ligand CCL17/TARC resulted only in variable enhancement of wound repair (Table 2). No repair responses could be observed in cultured keratinocytes when an ‘irrelevant’ (receptor-negative) ligand such as CXCL12 (CXCR4 ligand) was present (Table 2).

**Table 2:** Chemokines enhance wound repair in vitro

ng/ml	Endothelial cells				Fibroblasts				Keratinocytes			
	10	100	500	1000	10	100	500	1000	10	100	500	1000
CCL1/I-309	40%	40%	60%	n.d.	n.d.	n.d.	n.d.	n.d.	n.d.	n.d.	n.d.	n.d.
CCL3/MIP-1α	×	×	×	×	–	–	–	–	×	×	×	×
CCL11/eotaxin	–	50%	100%	80%	30%	30%	50%	–	40%	40%	70%	60%
CCL17/TARC	–	–	–	n.d.	50%	60%	50%	–	–	70%	40%	30%
CCL20/MIP-3α	–	–	–	n.d.	×	×	×	×	100%	80%	60%	30%
CCL27/CTACK	–	40%	100%	80%	100%	70%	50%	80%	–	–	–	n.d.
CXCL8/IL-8	40%	–	–	n.d.	×	×	×	×	50%	80%	70%	80%
CXCL10/IP-10	–	–	–	n.d.	×	×	×	×	–	20%	80%	100%

Wound repair of single path wounds in confluent cultures of human primary dermal microvascular endothelial cells, dermal fibroblasts and epidermal keratinocytes in the presence of indicated chemokines was monitored 12–18 h after injury. Percentage of wound closure was normalized to medium alone. Experiments were performed in triplicates and representative results of three different donors are shown. (−, closure of wound under 10%; n.d., not determined; ×, no receptor expressed for this ligand)

Cultured dermal fibroblasts express CCR3, CCR4 and CCR10. Accordingly, CCL11 (CCR3 ligand), CCL17 (CCR4 ligand) and CCL27 (CCR10 ligand) induced significant migration of cells into single path wounds leading to enhanced wound repair while ‘irrelevant’ chemokines such as CCL3 (CCR1 and CCR5 ligand) failed to induce responses (Table 2).

Cultured dermal microvascular endothelial cells, finally, that were shown to express the broadest chemokine receptor expression pattern (CCR3, CCR4, CCR6, CCR8, CCR9, CCR10, CXCR1, CXCR2, CXCR3) among structural cells of the skin presented markedly enhanced wound repair responses after stimulation with CCL1 (CCR8 ligand), CCL11 (CCR3 ligand) and CCL27 (CCR10 ligand) (Table 2). Variable results were obtained with CXCL8 (CXCR1 and CXCR2 ligand). Although dermal microvascular endothelial cells expressed significant levels of CCR6 and CXCR3 on their cell surface, no enhancement of wound repair was observed with their ligands CCL20 and CXCL10 (Table 2). Notably, instead of promoting endothelial migration and angiogenesis, CXCR3 ligands have recently been associated with angiostasis.

Finally, responsiveness of structural cells towards relevant chemokine ligands was pertussis toxin-sensitive, indicating the necessity of G(i) alpha protein-coupled receptor signaling for chemokine-induced wound repair in vitro.

### The chemokine network in human cutaneous wound healing

**Fig. 5:**
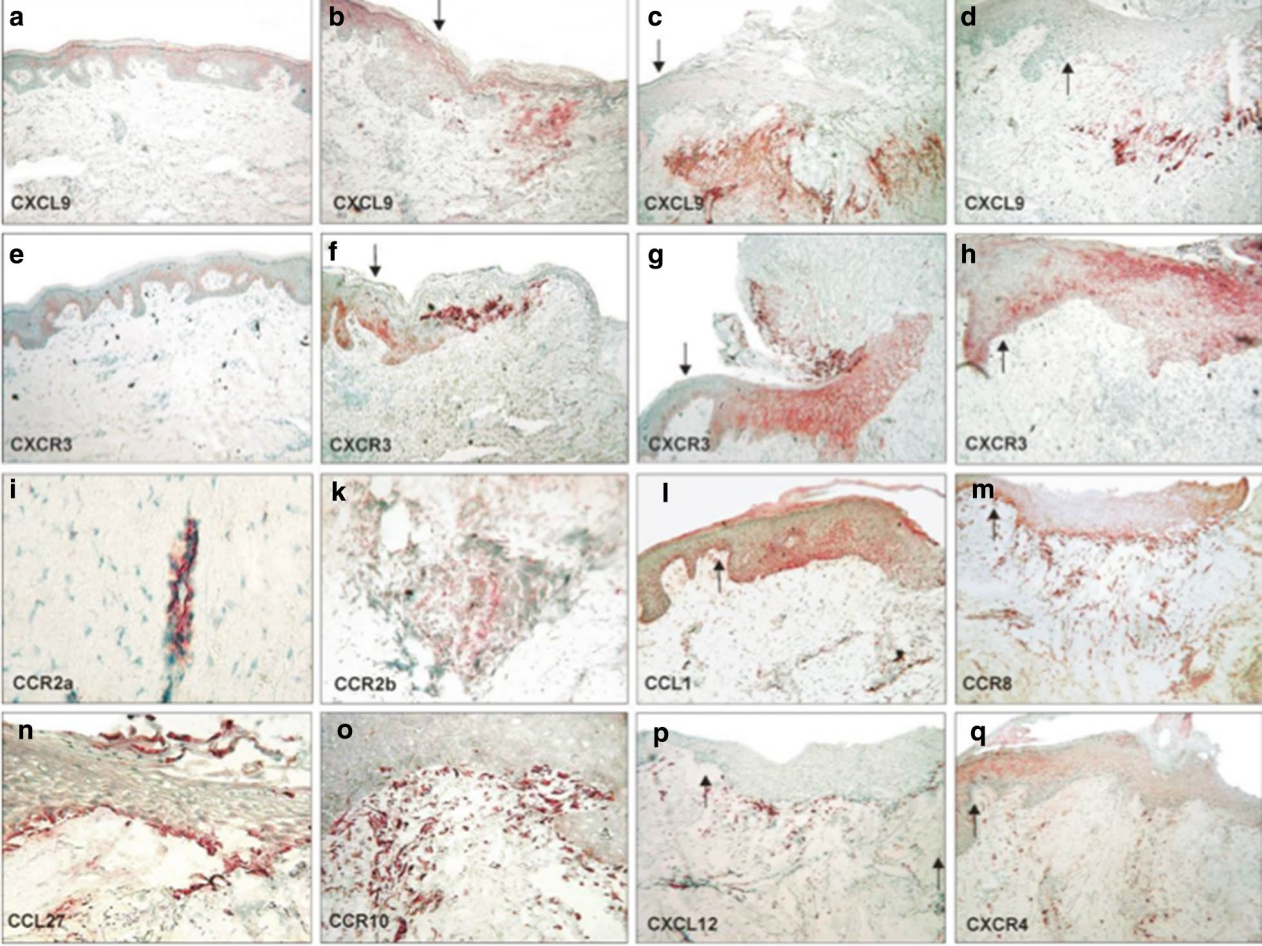
Chemokine expression during cutaneous wound healing. Immunohistochemical analyses of chemokine expression in healthy versus wounded human skin. Kryo sections (6 μm) of healthy and skin undergoing secondary wound healing (2, 5, 7 and 12 days after wounding) were stained with antibodies directed against CXCL9/Mig (**a**–**d**), CXCR3 (**e**–**h**), CCR2α (**i**), CCR2β (**k**), CCL1/I-309 (**l**), CCR8 (**m**), CCL27/CTACK (**n**), CCR10 (**o**), CXCL12/SDF-1α (**p**), and CXCR4 (**q**). **a**, **e** Healthy skin; **b**, **f**, **i**, day 2 after injury; **c**, **g**, **l**, **q** day 7 after injury; **d**, **h**, **k**, **m**–**p**, day 12 after injury; magnification 100 or ×250. The wound edge points to the right border of the picture. The arrow marks the beginning of the neo-epidermis. Representative results of four different donors

To confirm findings in mouse and in vitro observations with human structural cells, we studied the kinetics of human secondary wound healing in vivo (Fig. 5; Table 3). Immunohistochemical analyses of chemokine ligand and receptor expression in skin biopsies taken from wound edges on day 0, 2, 5, 7, 10, and 12 after radical surgery and secondary wound healing showed that structural cells as well as skin-infiltrating leukocytes are sources of abundant chemokine production (Fig. 5; Table 3). In addition to resident and skin-infiltrating leukocytes, structural cells also expressed and regulated chemokine receptor expression during the healing process. To identify the cellular origin of chemokine ligand and receptor expression, serial sections were stained with anti-CD3 to identify lymphocytes, anti-CD68 for skin- infiltrating monocytes, macrophages and dendritic cells or anti-CD31 as a marker for endothelial cells. Keratinocyte- and fibroblast-derived chemokine and chemokine receptor expression was determined by their characteristic phenotype and anatomical location.

**Table 3:** Immunohistochemical analysis of chemokine expression in healthy skin compared with injured human skin

*Genes*		Normal skin	Day 2 p.o.	Day 5 p.o.	Day 9 p.o.	Day 12 p.o.	*Genes*		Normal skin	Day 2 p.o.	Day 5 p.o.	Day 9 p.o.	Day 12 p.o.
CCR1	Neutrophil	−	−	−	−	−	CCL1	Neutrophil	−	−	−	−	−
	Macrophage	+	++	++	+	+	*I*-*309*	Macrophage	−	−	−	−	−
	Lymphocyte	−	−	−	−	−		Lymphocyte	−	−	−	−	−
	Keratinocyte	−	−	−	−	−		Keratinocyte	+	+	+	+	+
	Fibroblast	−	−	−	−	−		Fibroblast	−	−	−	−	−
	Endothelial cell	−	−	−	−	−		Endothelial cell	++	+++	+++	+++	++
CCR2α	Neutrophil	−	−	−	−	−	CCL4	Neutrophil	−	−	−	−	−
	Macrophage	−	−	−	−	−	*MIP*-*1β*	Macrophage	+	++	++	+	+
	Lymphocyte	−	−	−	−	−		Lymphocyte	−	−	−	−	−
	Keratinocyte	−	−	−	−	−		Keratinocyte	−	−	−	−	−
	Fibroblast	−	−	−	−	−		Fibroblast	−	−	−	−	−
	Endothelial cell	+	++	++	+	+		Endothelial cell	+	++	+	+	+
CCR2β	Neutrophil	−	−	−	−	−	CCL11	Neutrophil	−	−	−	−	−
	Macrophage	+	++	++	+	+	*eotaxin*	Macrophage	−	−	−	−	−
	Lymphocyte	−	+	+	+	+		Lymphocyte	−	−	+	+	+
	Keratinocyte	−	−	−	−	−		Keratinocyte	−	−	−	−	−
	Fibroblast	−	−	−	−	−		Fibroblast	+	++	++	+++	++
	Endothelial cell	−	−	−	−	−		Endothelial cell	+	+	+++	+++	+
CCR3	Neutrophil	−	−	−	−	−	CCL17	Neutrophil	−	−	−	−	−
	Macrophage	−	−	−	−	−	*TARC*	Macrophage	−	−	−	−	−
	Lymphocyte	−	+	++	++	+		Lymphocyte	−	−	−	−	−
	Keratinocyte	+	+	+	+	+		Keratinocyte	+	++	++	+	+
	Fibroblast	−	+	++	++	+		Fibroblast	−	−	−	−	−
	Endothelial cell	+	+	++	++	+		Endothelial cell	+	+++	+++	++	+
CCR4	Neutrophil	−	−	−	−	−	CCL18	Neutrophil	−	−	−	−	−
	Macrophage	−	−	−	−	−	*DC*-*CK1*	Macrophage	−	+	+	+	−
	Lymphocyte	−	−	++	++	+		Lymphocyte	−	−	−	−	−
	Keratinocyte	+	+	+	+	+		Keratinocyte	+	++	++	++	+
	Fibroblast	−	−	+	−	−		Fibroblast	−	−	−	−	−
	Endothelial cell	+	+	++	++	+		Endothelial cell	−	−	−	−	−
CCR5	Neutrophil	−	−	−	−	−	CCL20	Neutrophil	−	−	−	−	−
	Macrophage	−	−	−	−	−	*MIP-3α*	Macrophage	−	−	−	−	−
	Lymphocyte	−	−	−	−	−		Lymphocyte	−	−	−	−	−
	Keratinocyte	−	−	−	−	−		Keratinocyte	+	++	+++	+++	++
	Fibroblast	−	−	−	−	−		Fibroblast	−	−	−	−	−
	Endothelial cell	−	−	−	−	−		Endothelial cell	−	−	−	−	−
CCR6	Neutrophil	−	−	−	−	−	CCL24	Neutrophil	−	−	−	−	−
	Macrophage	−	−	−	−	−	*eotaxin-2*	Macrophage	−	−	−	−	−
	Lymphocyte	−	−	−	−	−		Lymphocyte	−	−	−	−	−
	Keratinocyte	++	++	++	++	++		Keratinocyte	−	−	−	−	−
	Fibroblast	−	−	−	−	−		Fibroblast	−	−	−	−	−
	Endothelial cell	−	++	++	++	++		Endothelial cell	+	+	++	++	+
CCR8	Neutrophil	−	−	−	−	−	CCL27	Neutrophil	−	−	−	−	−
	Macrophage	+	++	++	+	+	*CTACK*	Macrophage	−	−	−	−	−
	Lymphocyte	−	+	+	+	+		Lymphocyte	−	−	−	−	−
	Keratinocyte	−	−	−	−	−		Keratinocyte	+	++	+	+	+
	Fibroblast	−	−	−	−	−		Fibroblast	−	−	−	−	−
	Endothelial cell	+	+	++	++	+		Endothelial cell	−	−	−	−	−
CCR10	Neutrophil	−	−	−	−	−	CXCL8	Neutrophil	−	−	−	−	−
	Macrophage	−	−	−	−	−	*IL-8*	Macrophage	−	−	−	−	−
	Lymphocyte	−	+	++	++	++		Lymphocyte	−	−	−	−	−
	Keratinocyte	−	−	−	−	−		Keratinocyte	+	+++	++	++	+
	Fibroblast	−	−	−	−	−		Fibroblast	−	−	−	−	−
	Endothelial cell	++	+	+++	++	++		Endothelial cell	+	++	++	++	+
CXCR1	Neutrophil	−	−	−	−	−	CXCL9	Neutrophil	−	−	−	−	−
	Macrophage	−	−	−	−	−	*Mig*	Macrophage	−	+++	++	+	−
	Lymphocyte	−	−	−	−	−		Lymphocyte	−	−	−	−	−
	Keratinocyte	+	+	+	+	+		Keratinocyte	+	+	+	+	+
	Fibroblast	−	−	−	−	−		Fibroblast	−	−	−	−	−
	Endothelial cell	+	++	++	++	+		Endothelial cell	−	−	−	−	−
CXCR2	Neutrophil	−	+	+	−	−	CXCL10	Neutrophil	−	−	−	−	−
	Macrophage	−	−	−	−	−	*IP*-*10*	Macrophage	−	−	−	−	−
	Lymphocyte	−	−	−	−	−		Lymphocyte	−	−	−	−	−
	Keratinocyte	+	+	+	+	+		Keratinocyte	+	+	+	+	+
	Fibroblast	−	−	−	−	−		Fibroblast	−	−	−	−	−
	Endothelial cell	−	++	++	−	−		Endothelial cell	−	+	++	+	−
CXCR3	Neutrophil	−	−	−	−	−	CXCL12	Neutrophil	−	−	−	−	−
	Macrophage	−	−	−	−	−	*SDF-1α*	Macrophage	+	+++	+++	++	+
	Lymphocyte	−	−	−	−	−		Lymphocyte					
	Keratinocyte	+	++	+++	+	+		Keratinocyte	−	−	−	−	−
	Fibroblast	−	−	−	−	−		Fibroblast	−	−	−	−	−
	Endothelial cell	−	−	+	−	−		Endothelial cell	+	++	+++	+++	+
CXCR4	Neutrophil	−	−	−	−	−							
	Macrophage	+	+++	+++	+	+							
	Lymphocyte		+	+	+	+							
	Keratinocyte	+	+	+	++	++							
	Fibroblast	−	−	−	−	−							
	Endothelial cell	+	+	+	+	+							

Cryosections (6 µm) of skin samples, derived either from healthy subjects or from subjects who are going through an acne inversa treatment (2, 5, 9 and 12 days after trauma) with secondary wound healing were stained with antibodies directed against the listed chemokine and chemokine receptors. Representative results from four different donors. +, weak; ++, moderate; +++, strong staining

In normal skin, CCL27 represented the dominant chemokine expressed by basal keratinocytes of the epidermis. Other homeostatically but weakly keratinocyte-expressed chemokines included CCL1 (basal keratinocytes), CCL17 (suprabasal keratinocytes), CCL18/DC-CK1 (suprabasal keratinocytes), CXCL8 (suprabasal keratinocytes), and CXCL10 (basal keratinocytes) (Table 3). Interestingly, the most prominent expression of CCL1 was associated with the basal layers of hair follicles. Furthermore, immunohistochemical analyses revealed that keratinocytes also express chemokine receptors constitutively. Basal keratinocytes of normal epidermis markedly expressed CXCR3 on their cell surface (Fig. 5e). During cell differentiation, keratinocytes appeared to lose CXCR3 but gained CXCR4 expression resulting in CXCR3^−^/CXCR4^+^ suprabasal keratinocytes in healthy skin (Fig. 5q). Similar to CXCR3, CCR6 was expressed at high levels in basal keratinocytes of healthy skin (Table 3). Other chemokine receptors which were expressed at lower levels by keratinocytes in normal skin were CCR3 (basal and suprabasal keratinocytes), CCR4 (suprabasal keratinocytes), and CXCR1 (suprabasal keratinocytes) (Table 3).

Next to keratinocytes, dermal endothelial cells also showed abundant homeostatic chemokine ligand (CCL1, CCL17, CCL24, CXCL12) and receptor (CCR2α, CCR3, CCR4, CCR8, CCR10, CXCR1, CXCR2, CXCR3) expression confirming our previous in vitro results.

After cutaneous injury, monocytes infiltrate the skin, get activated, and differentiate into macrophages and dendritic cells expressing CCR1, CCR2β, CCR6, CCR8, and CXCR4 (Fig. 5; Table 3). CD3-positive lymphocytes, which were present as early as 2 days after injury and reside in perivascular pockets demarcating the wound bed, expressed a variety of chemokine receptors including CCR3, CCR4, CCR6, CCR8, CCR10, CXCR3, and CXCR4 (Table 3).

Two days after wounding, keratinocytes of the wound edge migrate into the wound bed and start to form a neo-epidermis. Basal keratinocytes of the neo-epidermis are strong producers of CCL27 throughout the re-epithelialization period. Next to CCL27, basal keratinocytes of the neo-epidermis also show low levels of CCL1 expression (Fig. 5l, n). Overall weak expression of CXCL10 was detected in keratinocytes bordering or distant to the wound edge (Table 3). During resurfacing of the wound, keratinocytes migrate as a collective with basal cells leading the way. Interestingly, CCR6 and CXCR3 were also highly expressed in this subset of migrating basal keratinocytes (Table 3). Suprabasal keratinocytes of the neo-epidermis were also an abundant source of CCL18, CCL20 and CXCL8 and expressed low levels of CCR3, CCR4, and CXCR1 on their cell surface (Table 3). During tissue remodeling (> day 10), the neo-epidermis reorganized and started to show stratified receptor expression with CXCR3 on basal and strong CXCR4 expression on differentiating suprabasal keratinocytes (Fig. 5e–h; Table 3).

CD68^+^ leukocytes were seen at perivascular pockets delimiting the wound bed and in close proximity to the developing neo-epidermis. This population of skin infiltrating monocytes, activated macrophages and dendritic cells was an abundant source of CCL18, CXCL9, and CXCL12 (Fig. 5a–d, p; Table 3).

Starting from day 4 after injury, endothelial cells infiltrate the wound bed along provisional extracellular matrix. Wound-infiltrating endothelial cells expressed a broad spectrum of chemokine receptors, such as CCR3, CCR4, CCR6, CCR8, CCR10, CXCR1, CXCR2, CXCR3, and CXCR4 (Fig. 5; Table 3). CCR6 was the most dominant chemokine receptor expressed on endothelial cells after day 10 of wound healing (Table 3). Endothelial cells of the wound edge and those infiltrating the wound also produced a variety of chemokines including CXCL8, CXCL10, CXCL12, and CCL24 (Table 3). Although cultured microvascular endothelial cells did not express significant levels of CCR2 in vitro, antibodies raised against the splice variant CCR2α showed strong expression of CCR2 on endothelial cells (Fig. 5i; Table 3). An antibody directed against another isoform of CCR2 (CCR2β) showed a different staining pattern with dominant immunoreactivity on CD68^+^ leukocytes (Fig. 5k; Table 3). Another chemokine which has previously been shown to be expressed by endothelial cells, CCL17, was found to be expressed in human secondary wounds by keratinocytes and endothelial cells around day 5 (Table 3). CCR4, receptor of CCL17, was homeostatically expressed on suprabasal keratinocytes in healthy and wounded skin (Table 3). Furthermore, CCR4 could be detected on endothelial cells within the wound bed confirming in vitro findings with cultured microvascular endothelial cells (Additional file 1: Figure S3).

In summary, our findings in human secondary wound healing confirm both our initial observations in mice and in vitro results, and suggest several autocrine and heterocrine loops of chemokine-induced cell activation and suggest a temporal–spatial chemokine ligand and receptor expression which may regulate biological processes including cell migration during cutaneous wound healing.

## Discussion

In recent years, several mediators including cytokines and chemokines have been suggested to participate in the process of wound healing [1, 2, 17, 18]. So far, most reports have mainly focused on the role of single molecules or single ligand–receptor combinations and comprehensive studies are missing. The rapid discovery of the complete chemokine superfamily offers the opportunity to take a “global view” in order to identify all family members of a particular molecular family involved in a biological process.

In the present study, we systematically investigated the potential role of several members of the chemokine superfamily in cutaneous wound healing, characterized their cellular origin as well as their regulation by proinflammatory mediators and their putative function. We have performed the analysis of the chemokine network on the mRNA- and also on the protein level, and secondly we investigated mouse and human wound healing models in vitro and in vivo. Our results suggest a complex interplay of structural cells via chemokine ligand–receptor interactions and we identified several members of the chemokine superfamily as being among the most highly regulated genes during cutaneous wound healing.

Cutaneous wound healing occurs via sequential overlapping phases starting with the injury and consecutive with the hemostasis followed by inflammation, re-epithelization, granulation tissue- formation and finally tissue remodeling. Within hours after tissue injury, neutrophils are recruited to cleanse the wound of foreign, potentially infectious particles [1, 2]. Neutrophil infiltration into the wound is followed by an influx of monocytes which differentiate to activated macrophages and release an array of growth factors such as platelet-derived growth factor (PDGF) and vascular endothelial growth factor (VEGF) together with chemoattractants initiating the formation of granulation tissue [1, 2]. Among the leukocyte subsets infiltrating cutaneous wounds, macrophages and macrophage-derived factors have been shown to play a pivotal role in the transition from inflammation to tissue repair since macrophage-depleted animals exhibit defective wound repair [4, 5]. In this phase, a plethora of cytokines along with chemokines is released. Chemokines such as CCL2, CCL3, CCL4 and CCL5 are candidates to mediate monocyte/macrophage recruitment to wound sites via CCR1, 2, 3 and 5 [2, 3, 26]. Our findings confirmed strong CCL2, CCL3 and CCL4 expression during the inflammatory phase of mouse wound healing and show matching temporal expression of their main receptors CCR1 and CCR2 on wound-associated CD68^+^ macrophages [2]. Macrophages and dendritic cells are also an abundant source of chemokine ligands during cutaneous wound healing. In humans, macrophages and dendritic cells at wound sites secrete high levels of CCL18, CXCL9, 12 and CXCL22. In fact, previous findings in arteriosclerosis and lung diseases support strong CCL18 expression by lesional macrophages and dendritic cells [27–29].

Our observations also suggest that other monocyte/macrophage chemoattractants such as CCL1 and CXCL12 may be involved in the recruitment of CCR8/CXCR4-positive macrophages/dendritic cells during the inflammation and the transition to the tissue formation phase of cutaneous wound healing. However, this is in contrast to the more recent results of Zheng L et al. who described CXCL8 and CCL2 as important chemokines in the inflammation phase and hemostasis phase produced by macrophages [30]. Our immunohistochemical analysis of acne inversa patients showed no significant expression of CXCL8 in macrophages but particularly in keratinocytes and endothelial cells, with a high expression of CXCL8 after 2 days.

The influx of macrophages is followed by the infiltration of lymphocytes into the wound site around days [2, 3]. Lymphocytes remain in the demarcating zone for the entire wound healing process while the majority of neutrophils and macrophages disappear after the formation of granulation tissue. Strikingly, a large variety of lymphocyte chemoattractants such as CCL1, CCL2, CCL3, CCL4, CCL5, CCL7, CCL11, CCL18/DC-CK, CCL20, CCL24, CCL27, CXCL9, CXCL10, CXCL12, and XCL1 are present during the inflammation and tissue formation phase of wound healing. Correspondingly, a strong expression of CCR2, CCR3, CCR6, CCR8, CCR10, CXCR3 and CXCR4 can be detected on the surface of CD3^+^ lymphocytes at wound sites. In summary, the immune system provides multiple and redundant mechanisms to attract lymphocytes to sites of injury and tissue repair. Yet, their precise role during tissue formation and remodeling, besides representing sentinels of the immune system, is currently not well understood. In this context, the predominant presence of CD3^+^ lymphocytes in the perivascular pockets of sprouting blood vessels during cutaneous wound healing points toward a possible role in supporting endothelial cell proliferation, migration and invasion.

Tissue granulation and re-epithelialization of the wound bed are the next important processes during wound healing. Within days after injury, the phenotype of keratinocytes of the wound edge markedly changes. In fact, the cells reorganize their cytoskeleton, dissolve intercellular desmosomes, loose hemidesmosomal links between the epidermis and the dermis, and upregulate integrin receptors [1]. Altogether, these processes enable keratinocytes to migrate along viable tissue into the wound bed. However, the stimuli determining the migration of keratinocytes during the formation of a neo-epidermis have not been completely elucidated yet. The in vitro findings presented in this study show that human keratinocytes express a distinct set of functionally active chemokine receptors including CCR3, CCR4, CCR6, CXCR1, CXCR2, CXCR3 on their cell surface. Ligands to particular receptors significantly enhance keratinocyte migration and promote wound repair in vitro. Chemokine receptor expression by keratinocytes was confirmed in vivo during human secondary wound healing pointing to several important chemokine ligand–receptor combinations in the process of re- epithelialization.

In normal skin, basal keratinocytes express high levels of CXCR3 on their cell surface. Yet, CXCR3 expression of keratinocytes appears to be differentiation-dependent since suprabasal keratinocytes of the stratum spinosum or granulosum loose CXCR3 expression. In contrast, differentiating keratinocytes gain CXCR4 expression in vivo. Previously, immunohistochemical analyses of Charbonnier et al. suggested CCR6 expression by keratinocytes but the biological function remained unclear [31]. Here, we have shown that signaling through CCR6 can enhance keratinocyte wound repair in vitro.

In vitro, CCL27 markedly enhanced wound repair of endothelial cell and fibroblast indicating a role for CCL27–CCR10 interactions in tissue repair.

Approximately, 4 days after injury granulation tissue invades the wound bed. Fibroblasts start to produce a provisional matrix composed of fibrin, fibronectin, and hyaluronic acid, which is necessary to support cell ingrowth [1]. Cell movement into this matrix may require proteolytic functions which are provided by matrix-metalloproteinases such as plasmin, plasminogen activator, collagenase, gelatinase A or stromelysin, which are markedly upregulated between days 2 and 5 and may cleave a path for cell migration. Previous studies showed CCL2-induced matrix-metalloproteinase induction in dermal fibroblasts [32]. Although we could not detect significant CCR2 or CCR5 expression, both representing CCL2 ligands, on cultured fibroblasts, our in vitro findings show that primary dermal fibroblasts markedly express CCR3, CCR4, and CCR10 and respond with enhanced wound repair after stimulation with their ligands [33]. Notably, a broad array of CCR3 ligands, including CCL5, CCL7, CCL11 and CCL24, are upregulated in cutaneous wound healing suggesting a role for these ligand–receptor interactions in fibroblast biology and the tissue formation phase of the wound healing process.

Angiogenesis represents a complex process depending on extracellular matrix in the wound bed as well as migration and mitogenic stimulation of endothelial cells. During recent years, a variety of angiogenic factors such as basic fibroblast growth factor (bFGF), vascular endothelial growth factor (VEGF), transforming growth factor-β (TGF-β), angiogenin, angiotrofin, angiopoietin-1, thrombospondin, low oxygen and elevated lactic acid, have been linked to the induction of angiogenesis [1]. A current focus of chemokine research represents the investigation of the contribution of chemokines toward angiogenesis. Certain members of the CXC, e.g. CXCL8 and more recently also of the CC chemokine family induce endothelial cell migration in vitro and angiogenesis in vivo [3, 15, 16, 34–39]. Furthermore, angiogenic growth factors such as VEGF and bFGF have recently been shown to regulate chemokine receptor expression on endothelial cell suggesting a complex relationship of growth factors and chemokines in the regulation of angiogenesis [39]. In the present study, we show that human dermal microvascular endothelial cells express a distinct set of chemokine receptors, such as CCR3, CCR4, CCR6, CCR10, CXCR2, CXCR3 in vitro. Moreover, stimulation with the respective ligands (CCL1, CCL11, CCL27, CXCL8) enhanced wound injury repair of cultured endothelial cells. Another set of chemokines including CCL17, CCL20 and CXCL10 did not promote injury repair although their corresponding receptors (CCR4, CCR6 and CXCR3) are markedly expressed on endothelial cells. For other CXCR3 ligands, such as CXCL9, CXCL10 and CXCL11, there is accumulating evidence that they induce angiostasis through CXCR3 [40].

Bernardini et al. and Haque et al. demonstrated that the CCR8 ligand CCL1 binds to endothelial cells, stimulates chemotaxis and invasion of these cells, enhances human umbilical vein endothelial cell differentiation into capillary-like structures in vitro and induces angiogenesis in vivo [35–37]. Previously, it has been suggested that angiogenic ELR-motive-positive chemokines such as CXCL8 are expressed during the inflammation and tissue formation phase of wound healing and ELR-motive negative angiostatic chemokines such as CXCL10 appear later in the wound healing process dominating the tissue remodeling phase [3]. Our findings indicate that both angiogenic and angiostatic factors are present at all phases of cutaneous wound healing and suggest a more complex balance and regulation.

Taken together, chemokines and chemokine receptors are expressed in a distinct temporal and spatial manner during wound healing and play many different functions during wound healing processes. Our findings elucidate an important role for chemokines in the complex wound healing cascade and underscore the central paradigm that cutaneous injury begins with proinflammatory cytokine and chemokine production which results in leukocyte recruitment and subsequent growth factor production. In turn, growth factor production by leukocytes and structural cells combined with chemokine signalling will orchestrate the dynamic process of migration, invasion, and proliferation of parenchymal cells for tissue repair. Not much has been done in the clinic regarding the use of chemokines and their receptors as a basis for treatment. Chronic inflammation and alteration in angiogenesis can potentially be reduced or eliminated by interfering with proinflammatory or angiogenic chemokines and their receptors using small molecules that interfere with receptor function. To our knowledge, this study is the most comprehensive analysis of chemokine and chemokine receptor expression both in vivo and in vitro. In addition to the extensive analysis of the central structural and soluble network partners in wound healing, our work was able to elucidate previously unknown interactions between chemokine ligands and chemokine receptors. We were able to gain information on a faster wound healing through the use of specific chemokines and we revealed patterns of expression that have not been described so far. Especially ligand–receptor signaling through CCR6 can enhance keratinocyte wound repair in vitro. CCL27 markedly enhanced wound repair of endothelial cell and fibroblast indicating an important role for CCL27–CCR10 interactions in tissue repair.

Nevertheless, there are some limitations to this study, which are based on the fact that a very special patient collective of the so-called Acne inversa was investigated. To support the results of our work, the number of samples should be increased and different patient groups should be examined in subsequent studies. The selection of the test mice can also contribute to the results. We have only examined female BALB/c mice in our work. However, recently published studies suggest that wound healing in female BALB/c mice may differ from male mice [41]. To achieve a positive therapeutic effect, the exact interaction and influence of certain parts of this complex wound healing network must be examined in following work.

In conclusion, our findings enhance the understanding of the underlying mechanisms for cutaneous wound healing and may impact the development of novel therapeutic strategies for the treatment of wound healing disorders such as chronic ulcers.

## Additional files

**Additional file 1: Figure S1.** Human primary keratinocytes expressing CCR4, CCR6, CCR9, CXCR1 and CXCR3 on their surface. Flow cytometric analysis of chemokine receptor repertoire in cultured human primary keratinocytes. Representative results from one of at least three different donors.

**Additional file 2: Figure S2.** Human primary dermal fibroblasts expressing CCR3, CCR4 and CCR10 on their surface. Flow cytometric analysis of chemokine receptor repertoire in cultured human primary dermal fibroblasts. Representative results from one of at least three different donors.

**Additional file 3: Figure S3.** Human primary dermal microvascular endothelial cells expressing CCR3, CCR4, CCR6, CCR8, CCR9, CCR10, CXCR1, CXCR2, CXCR3 and CXCR4 on their surface. Flow cytometric analysis of chemokine receptor repertoire in cultured human primary dermal microvascular endothelial cells. Representative results of one of at least three different donors.
