# Supplementary figures and images for: Chemokine ligand–receptor interactions critically regulate cutaneous wound healing
Source: Eur J Med Res. 2018 Jan 16;23:4. doi: 10.1186/s40001-017-0299-0 (PMC5771017; doi:10.1186/s40001-017-0299-0)

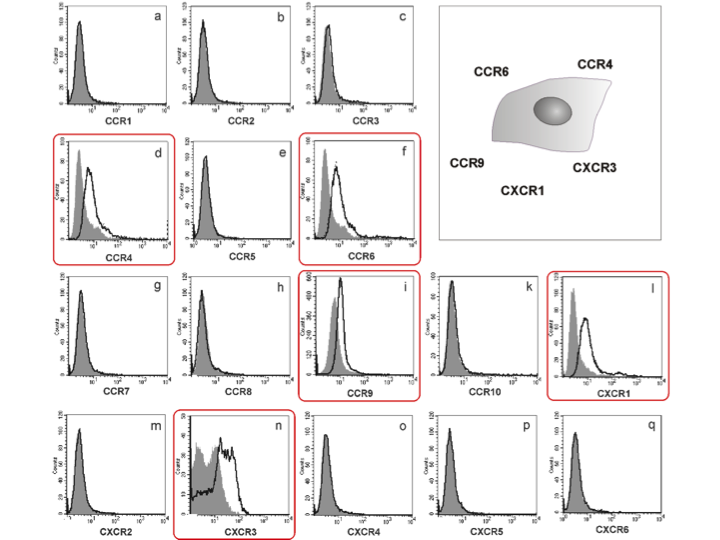

Supplement: Supplementary file 1 — Additional file 1: Figure S1. Human primary keratinocytes expressing CCR4, CCR6, CCR9, CXCR1 and CXCR3 on their surface. Flow cytometric analysis of chemokine receptor repertoire in cultured human primary keratinocytes. Representative results from one of at least three different donors. [file 40001_2017_299_MOESM1_ESM.tif]

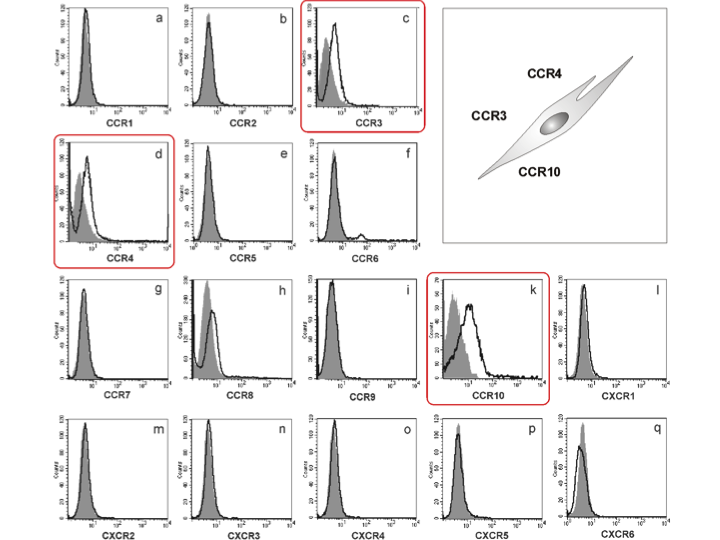

Supplement: Supplementary file 2 — Additional file 2: Figure S2. Human primary dermal fibroblasts expressing CCR3, CCR4 and CCR10 on their surface. Flow cytometric analysis of chemokine receptor repertoire in cultured human primary dermal fibroblasts. Representative results from one of at least three different donors. [file 40001_2017_299_MOESM2_ESM.tif]

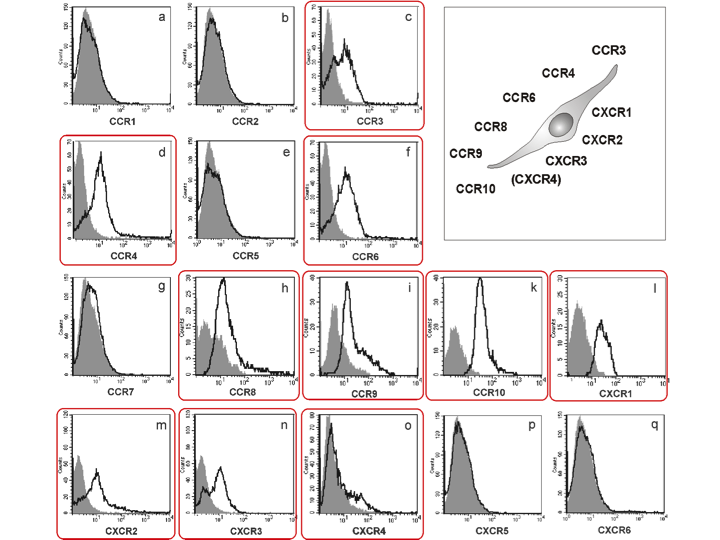

Supplement: Supplementary file 3 — Additional file 3: Figure S3. Human primary dermal microvascular endothelial cells expressing CCR3, CCR4, CCR6, CCR8, CCR9, CCR10, CXCR1, CXCR2, CXCR3 and CXCR4 on their surface. Flow cytometric analysis of chemokine receptor repertoire in cultured human primary dermal microvascular endothelial cells. Representative results of one of at least three different donors. [file 40001_2017_299_MOESM3_ESM.tif]
